# Context-dependent effects of SOM neurons to enhance the responses of corticocollicular neurons to repetitive sounds

**DOI:** 10.1016/j.isci.2026.115536

**Published:** 2026-03-30

**Authors:** Philip T.R. Bender, Mason McCollum, Emma Trate, Kaitlin Bainer, Rayli Ruby, Hui Li, Charles T. Anderson

**Affiliations:** 1 Department of Neuroscience, Rockefeller Neuroscience Institute, West Virginia University School of Medicine, Morgantown, WV 26506, USA

**Keywords:** neuroscience, systems neuroscience, sensory neuroscience

## Abstract

Stimulus-specific adaptation is a key feature of sensory systems, where the repeated presentation of a stimulus results in diminishing neuronal responses specifically to that stimulus, but not to a different stimulus. Cortical neurons display varying degrees of stimulus-specific adaptation, with cortical circuits shaping the dynamics of these responses. Here, we use calcium imaging and optogenetic approaches to assess the contributions of SOM and PV neurons to stimulus-specific adaptation in the mouse auditory cortex. We find opposing and complementary effects of SOM and PV neurons on the adaptation of principal neurons which manifest as context-dependent SOM neuron-mediated enhancement and PV-neuron mediated suppression of responses that depend on the temporal repetition rate of the sounds. Our results suggest that SOM and PV neurons can engage in multiple operating regimes that exist in a continuum during sensory processing.

## Highlights

• Corticocollicular (CCol) neurons demonstrate stimulus-specific adaptation
• Optogenetic inactivation of SOM neurons suppresses CCol neuron responses
• SOM neuron enhancement of CCol neuron responses is context-dependent

## Graphical abstract

**Figure:**
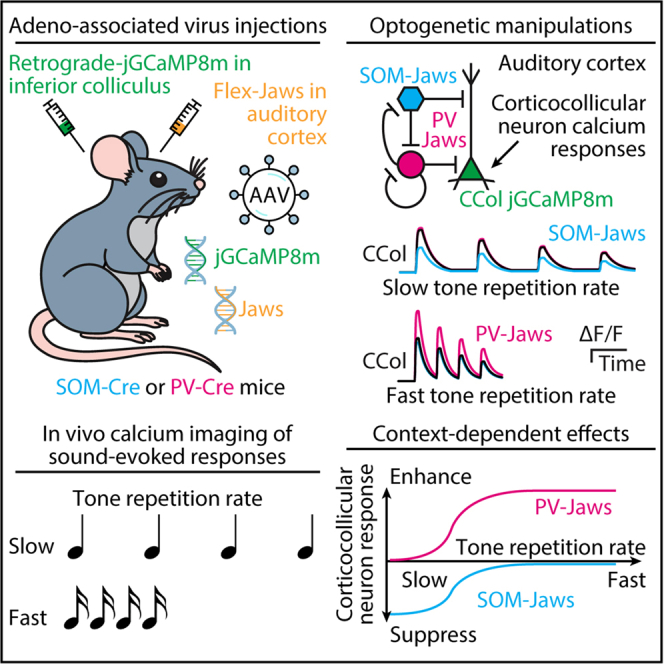

## Introduction

Sensory processing depends on accurately incorporating and responding to dynamic environmental cues in an ongoing process. A hallmark of many sensory systems is their ability to suppress responses to common or predictable environmental information while remaining sensitive to novel or unexpected information.^1^^,^^2^^,^^3^^,^^4^^,^^5^^,^^6^^,^^7^^,^^8^^,^^9^^,^^10^ In neurons, this phenomenon manifests as stimulus-specific adaptation in which a repeated stimulus results in a sequential reduction in neuronal responses over time, but a deviant stimulus will still elicit a robust response from the same neurons.^1^^,^^4^^,^^5^^,^^6^^,^^10^^,^^11^^,^^12^^,^^13^^,^^14^^,^^15^ The temporal pattern of the standard and deviant stimuli is a crucial factor in determining whether adaptation occurs, because events that occur too far apart from each other in time, or that are too distinct from each other do not elicit neuronal adaptation.^16^ Thus, none of the information that makes a stimulus a standard or a deviant is intrinsic to the stimulus but rather depends on the context in which the stimulus occurs. The rate of the build-up of adaptation to repeated stimuli, the temporal structure of the stimuli that allows this build-up to occur, and how quickly the adapted state is reset by the passage of time or the presence of a deviant stimulus are all important aspects for how ongoing context is accounted for by sensory systems, but these features remain poorly understood.

Corticocollicular neurons are a class of extratelencephalic corticofugal neurons which send long range axonal projections from the auditory cortex to subcortical regions, including the thalamus and inferior colliculus. They act as integrators of cortical activity, serving as a hub for information traveling from the lemniscal auditory pathway, through the cortex, and to the non-lemniscal auditory pathway for further processing.^17^^,^^18^ Many classes of cortical neurons exhibit stimulus-specific adaptation,^19^^,^^20^ and because corticocollicular neurons act as integrators of cortical information that receive inputs from many of these classes of excitatory and inhibitory neurons, they are uniquely poised to incorporate and transform these various signaling modalities to refine auditory processing. Because stimulus-specific adaptation is primarily a feature of non-lemniscal auditory processing,^21^^,^^22^^,^^23^^,^^24^^,^^25^^,^^26^ it is likely that corticocollicular neurons serve an important role in combining ascending auditory information from the lemniscal pathway with context-dependent processing from the non-lemniscal pathway that is crucial for stimulus-specific adaptation throughout the auditory system.

Multiple views about the roles of auditory cortex interneurons in shaping stimulus-specific adaptation have emerged,^14^^,^^27^ with a major focus on understanding the roles of the two most prevalent types of these GABAergic neurons: parvalbumin-expressing (PV) and somatostatin-expressing (SOM) neurons.^28^ These classes of neurons account for ∼80% of the inhibitory interneurons in the cortex and they powerfully shape the responses of principal neurons and of each other.^28^ Various motifs have been described that account for the effects of SOM and PV neurons on the sensory processing of principal neurons. In one proposed operating regime, cortical circuits act as an inhibition stabilized network, whereby reductions in inhibitory activity lead to a rapid increase of inhibition via the increased recurrent recruitment of inhibitory neurons.^29^^,^^30^^,^^31^ An alternative cortical operating regime engages disinhibitory hierarchies that exist between different types of interneurons, where PV neurons inhibit PV, SOM, and principal neurons; while SOM neurons inhibit PV and principal neurons, but not other SOM neurons.^32^^,^^33^ In this view, changes in principal neuron activity can be achieved by monosynaptic inhibitory signaling, or via polysynaptic disinhibitory interactions from SOM neurons to PV neurons to principal neurons. These different operating regimes can exist in parallel, and these aspects of cortical circuits can be engaged during the processing of repetitive and deviant sounds to shape the adaptation and deviance detection of principal neurons.^14^^,^^27^

Here, to understand how specific classes of cortical neurons support stimulus-specific adaptation over different timescales, we performed calcium imaging from different types of cortical neurons while using optogenetic approaches to suppress the activity of specific inhibitory cortical interneurons. We find that SOM interneurons serve to enhance the sound-evoked responses of corticocollicular principal glutamatergic neurons to repeated stimuli at slow tone repetition rates (one and two Hz tone repetition rates). In contrast we find that PV interneurons serve to suppress the sound-evoked responses of these same principal neurons, but only at higher tone repetition rates. These results suggest that there are complementary and cooperative roles for SOM neurons and PV neurons working in concert to shape stimulus-specific adaptation in the mouse auditory cortex. Our findings suggest that cortical circuits engage different aspects of disinhibition and inhibition stabilization operating regimes to shape neuronal function.

## Results

### Corticocollicular neurons display stimulus-specific adaptation

**Figure 1:**
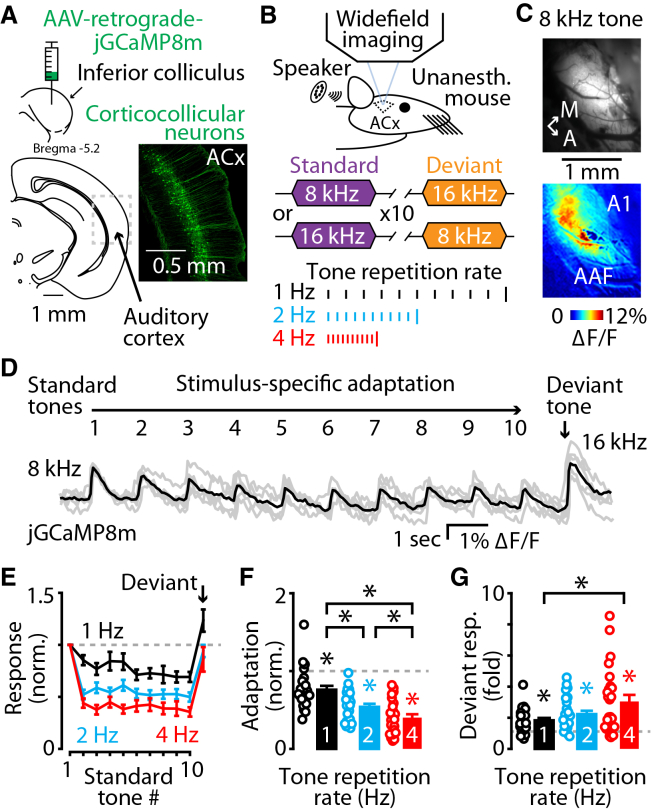
Corticocollicular neurons exhibit stimulus-specific adaptation (A) Top: cartoon showing the stereotaxic injection scheme for expressing the genetically encoded fluorescent calcium sensor jGCaMP8m in corticocollicular neurons in the auditory cortex of mice via retrograde AAV transfection. Bottom: schematic and example image of an acute brain slice showing the auditory cortex (ACx) with jGCaMP8m-expressing corticocollicular neurons shown in green. Scale bars: 0.5 mm. (B) Top: cartoon showing the experimental setup for *in vivo* widefield calcium imaging in unanesthetized mice. Middle: schematic representation of the two sound frequencies (8 kHz and 16 kHz) used as standard (purple) and deviant (orange) tones. Bottom: schematic representation of the presentation of the eleven-tone repeated sound auditory stimulus trains of ten standard tones (short lines) followed by a deviant tone (tall line) at three different tone repetition rates: 1 Hz (black), 2 Hz (blue), and 4 Hz (red). (C) Image of jGCaMP8m fluorescence from the cortical surface through a craniotomy over the auditory cortex. Bottom: example heatmap of the changes in jGCaMP8m fluorescence in response to an 80 dB SPL 8 kHz tone presented to the animal, highlighting the primary auditory cortex (A1) and the anterior auditory field (AAF). Scale bars: 1 mm. (D) Example averaged jGCaMP8m-mediated fluorescence widefield responses of corticocollicular neurons to the 1 Hz tone repetition rate repeated sound paradigm (black) with the individual recordings (gray). (E) Average jGCaMP8m-mediated widefield response to the repeated sound paradigm in B, showing adaptation to the standard tones and enhanced response to the deviant tone, indicative of stimulus-specific adaptation of corticocollicular neurons. Shown for three tone repetition rates: 1 Hz tone repetition rate, 2 Hz tone repetition rate, and 4 Hz tone repetition rate normalized to the amplitude of the calcium fluorescence response to the first tone in the train (1 Hz: *n* = 25 recordings from 20 mice. 2 Hz: *n* = 25 recordings from 20 mice. 4 Hz: *n* = 25 recordings from 20 mice). Color scheme as in (B). (F) Adaptation of corticocollicular neurons to repeated standard tones at different tone repetition rates. Average difference between the fluorescence response amplitude of the first tone (gray dashed line) and the fluorescence response amplitude of the tenth (last) standard tone in the stimulus train at 1 Hz, 2 Hz, and 4 Hz tone repetition rates (1 Hz: *p* = 4.46e−04, *n* = 25 recordings from 20 mice; one-sample Wilcoxon signed-rank test. 2 Hz: *p* = 2.28E−11, 25 recordings from 20 mice; one-sample *t* test. 4 Hz: *p* = 6.71e−13 *n* = 25 recordings from 20 mice; one-sample *t* test. 1 Hz v. 2 Hz: *p* = 4.03e−04; Wilcoxon signed-rank test. 1 Hz v. 4 Hz: *p* = 1.13e−04; Wilcoxon signed-rank test. 2 Hz v. 4 Hz: *p* = 0.0094; paired *t* test). Color scheme as in (B). (G) Responses of corticocollicular neurons to a deviant tone after a train of ten standard tones at different tone repetition rates. Average difference between the fluorescence response amplitude of the tenth (last) standard tone (gray dashed line) and the fluorescence response amplitude of the deviant tone in the stimulus train at 1 Hz, 2 Hz, and 4 Hz tone repetition rates (1 Hz: *p* = 1.30E−05, *n* = 25 recordings from 20 mice; one-sample *t* test. 2 Hz: *p* = 1.10E−05, 25 recordings from 20 mice; one-sample *t* test. 4 Hz: *p* = 1.10E−05, *n* = 25 recordings from 20 mice; one-sample Wilcoxon signed-rank test. 1 Hz v. 2 Hz: *p* = 0.0880; paired *t* test. 1 Hz v. 4 Hz: *p* = 0.0044; Wilcoxon signed-rank test. 2 Hz v. 4 Hz: *p* = 0.1094; Wilcoxon signed-rank test). Color scheme as in (B). Some data from Figures 1E–1G are also used in Figures 2D–2G, 3D–3G, S1B, S1C, S2E, S2F, S2H, S3B, and S3C. Asterisks indicate significant *p* values. Data are represented as mean ± SEM. See Table S1 for detailed statistics.

We began our investigation into the temporal dynamics of stimulus-specific adaptation by assessing the effects of temporal repetition rate on the adaptation to sound-evoked responses by cortical neurons. To perform this experiment, we induced the expression of jGCaMP8m into layer 5 corticocollicular neurons in the auditory cortex (Method details) and performed widefield imaging of calcium signals in the primary auditory cortex (A1) in head-fixed, chlorprothixene-sedated, unanesthetized mice (STAR Methods, Figures 1A and 1B). This retrograde viral approach selectively labels layer 5 corticocollicular neurons in the auditory cortex,^34^^,^^35^ which are also a subset of corticothalamic neurons that have a high density of axonal projections in the non-lemniscal regions of the medial geniculate thalamus.^34^^,^^36^^,^^37^^,^^38^ We presented ten tones of the same frequency (8 or 16 kHz, 100 msec duration, 5 msec cosine ramps) followed by a single tone of a different frequency (16 or 8 kHz) to quantify stimulus-specific adaptation and deviance detection of corticocollicular neurons (Figure 1B). Consistent with previous studies of the sound-evoked responses of these neurons,^35^^,^^39^ we observed stimulus-specific adaptation in neuronal responses using wide-field fluorescence imaging approaches (Figures 1A–1C). These responses became progressively smaller during repetitive presentation of standard tones and were larger for deviant tones (Figures 1D and 1E). Tone repetition rates of 2 and 4 Hz resulted in more adaptation of responses compared to 1 Hz tone repetition rates (Figure 1F), which were followed by significantly larger deviant tone response amplitudes at all tone repetition rates (Figure 1G). Together, these results show that the faster tone repetition rates resulted in larger amounts of stimulus-specific adaptation, suggesting that these different repetition rates may engage different aspects of cortical circuits important for context-dependent sound processing.

### SOM neurons enhance sound-evoked responses of corticocollicular neurons at lower tone-repetition rates

**Figure 2:**
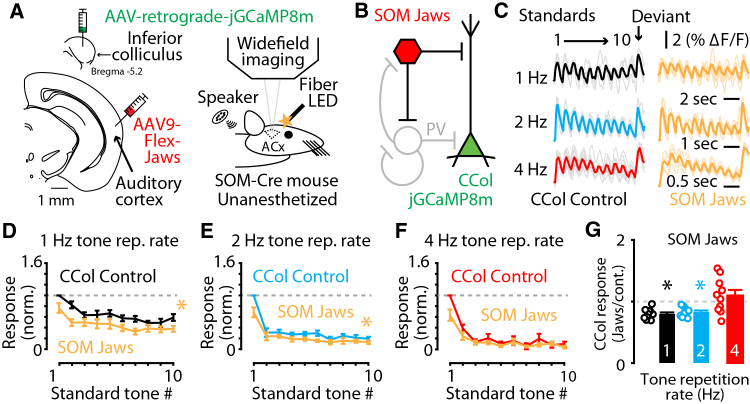
SOM neurons enhance corticocollicular neuron responses to trains of sounds at slower tone-repetition rates (A) Left: cartoon showing the stereotaxic injection scheme for expressing the genetically encoded fluorescent calcium sensor jGCaMP8m in corticocollicular neurons and the inhibitory, red-shifted opsin Jaws in SOM neurons in the auditory cortex of SOM-Cre mice. Right: cartoon showing the experimental setup for *in vivo* widefield calcium imaging and optical inactivation of Jaws-expressing SOM neurons in unanesthetized mice. (B) Cartoon circuit diagram showing Jaws-expressing SOM neurons (red) and jGCaMP8m-expressing corticocollicular neurons (green). (C) Example traces showing the individual (lighter lines) and average jGCaMP8m-mediated calcium fluorescence responses (darker lines) to the repeated sound paradigm at 1 Hz (black), 2 Hz (blue), and 4 Hz (red) tone repletion rate in both light-off control and light-on SOM neuron inactivation by Jaws (yellow). Deviant tone response is highlighted with an arrow. (D) Average corticocollicular neuron response to the 1 Hz tone repetition rate repeated sound paradigm standard tones in both light-off control (black) and light-on SOM neuron inactivation (yellow). Responses represented as light-on responses normalized to the corresponding light-off control first standard tone response: Response_n_/Control Response_1_. (1 Hz Light-off v. light-on: *p* = 0.0199, *n* = 9 recordings from five mice. Two-way repeated measures ANOVA with Tukey multiple comparisons). (E) Average corticocollicular neuron response to the 2 Hz tone repetition rate repeated sound paradigm standard tones in both light-off control (blue) and light-on SOM neuron inactivation (yellow). Responses represented as light-on responses normalized to the corresponding light-off control first standard tone response: Response_n_/Control Response_1_. (2 Hz light-off v. light-on: *p* = 0.0092, *n* = 7 recordings from four mice. Two-way repeated measures ANOVA with Tukey multiple comparisons). (F) Average corticocollicular neuron response to the 4 Hz tone repetition rate repeated sound paradigm standard tones in both light-off control (red) and light-on SOM neuron inactivation (yellow). Responses represented as light-on responses normalized to the corresponding light-off control first standard tone response: Response_n_/Control Response_1_. (4 Hz light-off v. light-on: *p* = 0.0656, *n* = 9 recordings from five mice. Two-way repeated measures ANOVA with Tukey multiple comparisons). (G) Bar plots showing the average change in corticocollicular neuron calcium fluorescence response amplitudes to all standard tones averaged together during light-on SOM neuron inactivation trials, normalized to the light-off trials, compared to no change (gray dashed line) at 1 Hz tone repetition rate (black), 2 Hz tone repetition rate (blue), and 4 Hz tone repetition rate (red). (1 Hz: *p* = 0.0219, *n* = 9 recordings from five mice. 2 Hz: *p* = 0.0071, *n* = 7 recordings from four mice. 4 Hz: *p* = 0.3123, *n* = 9 recordings from five mice. One-sample *t* tests). Asterisks indicate significant *p* values. Data are represented as mean ± SEM. See Table S1 for detailed statistics.

Because inhibitory neurons in the auditory cortex contribute to different aspects of adaptation and deviance detection,^14^^,^^20^^,^^27^ we hypothesized that inhibitory neuron activity could contribute to adaptation and deviance detection of corticocollicular neurons. To test this hypothesis, we investigated the effects of optogenetic inactivation of interneurons on the stimulus-specific adaptation of corticocollicular neurons. We used the inhibitory opsin Jaws, which is a red-light activated microbial chloride pump that hyperpolarizes neurons and inhibits action potential firing.^40^ We induced the expression of Jaws into SOM interneurons by injecting a Cre-dependent adeno-associated viral (AAV) construct in the auditory cortex of SOM-Cre mice (Method details), and we also injected a retrograde AAV construct encoding jGCaMP8m into the inferior colliculus in the same animals as above. After at least two weeks of recovery, we observed robust jGCaMP8m in corticocollicular neurons and Jaws expression in SOM neurons in the auditory cortex (Figures 2A and 2B). Mice were head-fixed as above and a fiber coupled LED (Method details) was positioned above the surface of the brain to deliver orange light to drive Jaws activation and inhibit SOM neuron activity in the auditory cortex (STAR Methods, Figure 2A). We performed interleaved light-off and light-on trials to assess the consequences of SOM neuron inactivation on corticocollicular sound-evoked responses (Figure 2C). SOM neuron inactivation caused a suppression of corticocollicular neuron sound-evoked responses for repeated standard tones presented at 1 and 2 Hz repetition rates, but had no effect on tones presented at a 4 Hz repetition rate (Figures 2D–2G). SOM neuron inactivation had no effect on the sound-evoked responses to the deviant tones, which were significantly larger than the responses to the last standard tone in both light-off and light-on conditions (Figure S1B). SOM neuron inactivation also did not change the relative amount of stimulus-specific adaptation we observed (Figure S1C). Opsin-based manipulations of interneurons can have counterintuitive effects on interneuron activity in cortical circuits, whereby inactivation of interneurons can lead to a net increase in the activity of those interneurons because of changes to the recruitment of feedforward and feedback inhibition in cortical circuits that operate as inhibition stabilized networks.^29^^,^^30^ If our results are associated with decreased SOM neuron activity during Jaws inactivation, this suggests that corticocollicular neuron activity is enhanced by SOM neuron activity during lower frequency tone repetition paradigms. To distinguish between potential explanations of our results, we performed similar Jaws and jGCaMP7b experiments to assess the effects of SOM neuron inactivation on the sound-evoked responses of SOM neurons. We expressed jGCaMP7b in SOM neurons via AAV injections into the auditory cortex of SOM-Cre mice. This labeling strategy resulted in ∼93% of SOM neurons expressing jGCaMP7b (Figures S2A–S2C). We then expressed both Jaws and jGCaMP7b in SOM neurons via dual AAV injections into the auditory cortex of SOM-Cre mice and performed similar widefield fluorescent experiments as above. We hypothesized that light-on trials would result in suppression of SOM neuron responses, thus verifying that Jaws suppresses SOM neurons *in vivo*. Consistent with our hypothesis, Jaws inactivation of SOM neurons resulted in decreased sound-evoked responses of SOM neurons at all tone repetition rates tested (Figures S2D–S2F). This result shows that SOM neuron sound-evoked responses are suppressed by activation of the inhibitory opsin Jaws in the auditory cortex and suggests that SOM neuron activity contributes to enhanced sound-evoked responses by corticocollicular neurons. In these mice, the effect of Jaws activation to suppress SOM neuron sound-evoked responses was not significantly different for the three tone repetition rates used (Figure S2F), which further supports the conclusion that the effects of SOM neurons on corticocollicular neurons do not follow a simple relationship in which less SOM neuron activity causes less corticocollicular neuron activity. Furthermore, in AAV sham animals where we used an AAV to induce GFP expression (instead of Jaws) in SOM neurons alongside jGCaMP7b expression in corticocollicular neurons (Figure S2G), there was no effect of light on the sound-evoked responses of corticocollicular neurons (Figure S2H). Therefore, the suppression of corticocollicular responses by SOM neuron inactivation is associated with the reduction of SOM neuron activity by Jaws during stimulus-specific adaptation. Together, these results suggest that SOM neurons serve to enhance the sound-evoked responses to lower repetition rate sounds.

### PV neurons suppress sound-evoked responses of corticocollicular neurons at higher tone-repetition rates

**Figure 3:**
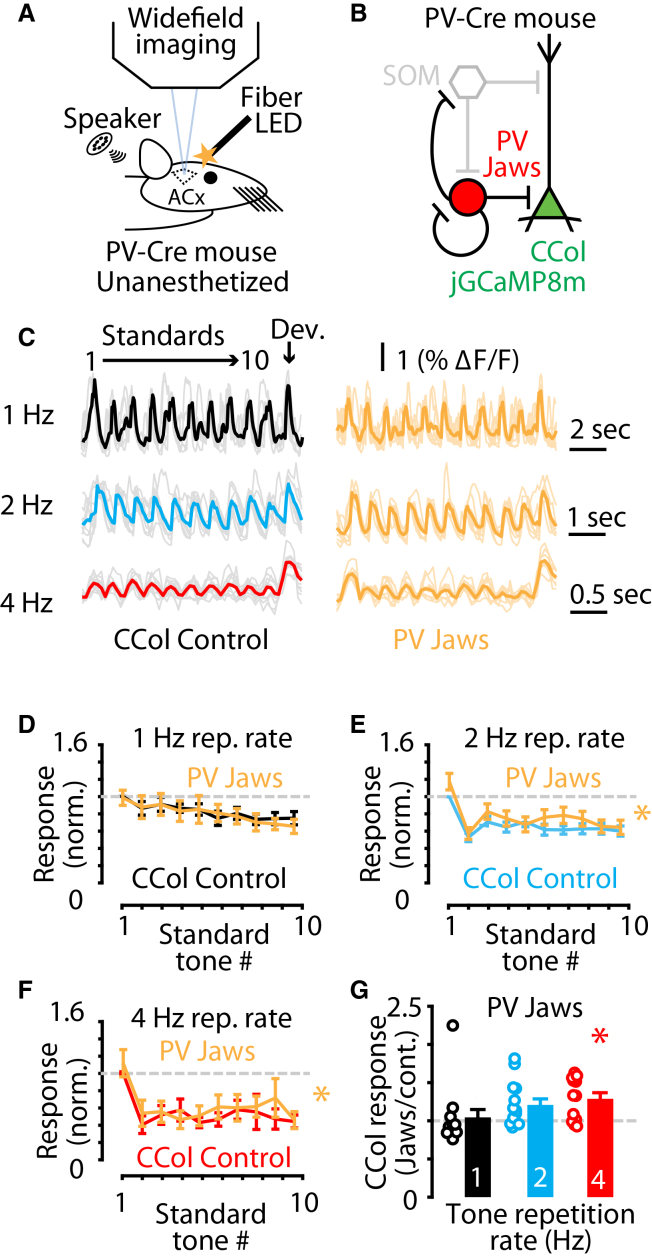
PV neurons suppress corticocollicular neurons responses to trains of sounds at faster tone-repetition rates (A) Cartoon showing the experimental setup for *in vivo* widefield calcium imaging and optical inactivation of Jaws-expressing PV neurons in unanesthetized mice. (B) Cartoon circuit diagram showing Jaws-expressing PV neurons (red) and jGCaMP8m-expressing corticocollicular neurons (green). (C) Example traces showing the individual (lighter lines) and average jGCaMP8m-mediated calcium fluorescence responses (darker lines) to the repeated sound paradigm at 1 Hz (black), 2 Hz (blue), and 4 Hz (red) tone repletion rate in both light-off control and light-on PV neuron inactivation by Jaws (yellow). Deviant tone response is highlighted with an arrow. (D) Average corticocollicular neuron response to the 1 Hz tone repetition rate repeated sound paradigm standard tones in both light-off control (black) and light-on PV neuron inactivation (yellow). Responses represented as light-on responses normalized to the corresponding light-off control first standard tone response: Response_n_/Control Response_1_. (1 Hz Light-off v. light-on: *p* = 0.9318, *n* = 12 recordings from seven mice. Two-way repeated measures ANOVA with Tukey multiple comparisons). (E) Average corticocollicular neuron response to the 2 Hz tone repetition rate repeated sound paradigm standard tones in both light-off control (blue) and light-on PV neuron inactivation (yellow). Responses represented as light-on responses normalized to the corresponding light-off control first standard tone response: Response_n_/Control Response_1_. (2 Hz light-off v. light-on: *p* = 0.0029, *n* = 14 recordings from eight mice. Two-way repeated measures ANOVA with Tukey multiple comparisons). (F) Average corticocollicular neuron response to the 4 Hz tone repetition rate repeated sound paradigm standard tones in both light-off control (red) and light-on PV neuron inactivation (yellow). Responses represented as light-on responses normalized to the corresponding light-off control first standard tone response: Response_n_/Control Response_1_. (4 Hz light-off v. light-on: *p* = 0.0090, *n* = 12 recordings from seven mice. Two-way repeated measures ANOVA with Tukey multiple comparisons). (G) Bar plots showing the average change in corticocollicular neuron calcium fluorescence response amplitudes during light-on PV neuron inactivation trials, normalized to the light-off trials, compared to no change (gray dashed line) at 1 Hz tone repetition rate (black), 2 Hz tone repetition rate (blue) and 4 Hz tone repetition rate (red). (1 Hz: *p* = 0.1763, *n* = 12 recordings from seven mice; one-sample Wilcoxon signed-rank test. 2 Hz: *p* = 0.0459, *n* = 14 recordings from eight mice; one-sample *t* test. 4 Hz: *p* = 0.0090, *n* = 12 recordings from seven mice; one-sample *t* test). Asterisks indicate significant *p* values. Data are represented as mean ± SEM. See Table S1 for detailed statistics.

The role of SOM neurons to enhance corticocollicular neuron sound-evoked responses could be a feature of inhibitory control of corticocollicular neurons, or it could arise from a specific role of SOM neurons in stimulus-specific adaptation. To distinguish between these possibilities, we performed experiments to assess the roles of PV neurons—another major class of inhibitory interneurons in the auditory cortex^41^^,^^42^^,^^43^ – on corticocollicular neuron sound-evoked responses. We hypothesized that PV neurons would function to suppress corticocollicular neuron responses to sounds. To test this hypothesis, we expressed Jaws in PV neurons using PV-Cre mice (Method details) and expressed jGCaMP8m in corticocollicular neurons in the same animals and then performed similar experiments using light-off and light-on trials (Figures 3A–3C). Consistent with our hypothesis, inactivation of PV neurons resulted in an increase in sound-evoked responses of corticocollicular neurons. However, in contrast to the inactivation of SOM neurons that resulted in suppressed corticocollicular neuron responses, PV neuron inactivation preferentially enhanced sound-evoked responses of corticocollicular neurons at higher tone repetition rates (Figures 3C–3G). PV neuron activity did not affect the response to deviant sounds in these same animals (Figure S3B). PV neuron inactivation also did not change the relative amount of stimulus-specific adaptation we observed (Figure S3C). Although the magnitude of the effect of PV neuron inactivation was smaller than the magnitude of SOM neuron inactivation (Figures 2G vs. 3G), the opposite direction of the effects on corticocollicular neuron sound-evoked responses (decrease vs. increase) suggests that corticocollicular neurons are differentially modulated by SOM vs. PV neurons. These results suggest that PV neurons function to suppress the sound-evoked responses of corticocollicular neurons, which is consistent with their well-established role of providing strong somatic inhibition of principal cells throughout the cortex.^44^ Together, these results suggest that the enhancing effects of SOM neurons on corticocollicular neuron sound-evoked responses are due to a SOM neuron-specific role during stimulus-specific adaptation and not a general feature of inhibitory neuron inputs to corticocollicular neurons in the auditory cortex.

### Complementary roles for SOM and PV neurons that contribute to corticocollicular neuron stimulus-specific adaptation

**Figure 4:**
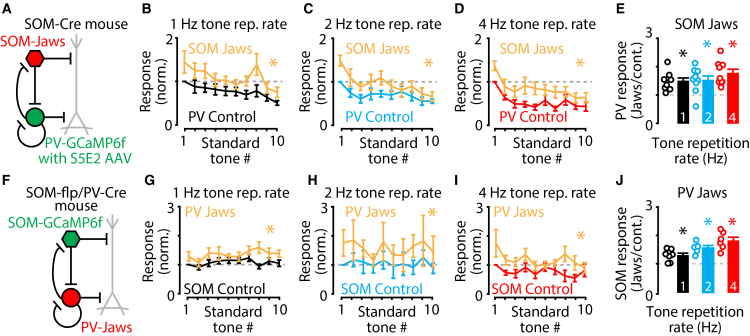
Disinhibitory connections from SOM neurons to PV neurons and from PV neurons to SOM neurons are present in the auditory cortex (A) Cartoon circuit diagram showing Jaws-expressing SOM neurons (red) and GCaMP6f-expressing PV neurons (green). (B) Average PV neuron response amplitudes to the 1 Hz tone repetition rate repeated sound paradigm standard tones in both light-off control (black) and light-on SOM neuron inactivation (yellow). Responses represented as light-on responses normalized to the corresponding light-off control first standard tone response: Response_n_/Control Response_1_. (1 Hz Light-off v. light-on: *p* = 0.0041, *n* = 9 recordings from six mice. Two-way repeated measures ANOVA with Tukey multiple comparisons). (C) Average PV neuron response amplitudes to the 2 Hz tone repetition rate repeated sound paradigm standard tones in both light-off control (blue) and light-on SOM neuron inactivation (yellow). Responses represented as light-on responses normalized to the corresponding light-off control first standard tone response: Response_n_/Control Response_1_. (2 Hz light-off v. light-on: *p* = 0.0197, *n* = 9 recordings from 6 mice. Two-way repeated measures ANOVA with Tukey multiple comparisons). (D) Average PV neuron response amplitudes to the 4 Hz tone repetition rate repeated sound paradigm standard tones in both light-off control (red) and light-on SOM neuron inactivation (yellow). Responses represented as light-on responses normalized to the corresponding light-off control first standard tone response: Response_n_/Control Response_1_. (4 Hz light-off v. light-on: *p* = 4.727e−4, *n* = 9 recordings from six mice. Two-way repeated measures ANOVA with Tukey multiple comparisons). (E) Bar plots showing the average change in PV neuron calcium fluorescence response amplitudes during light-on SOM neuron inactivation trials, normalized to the light-off trials, compared to no change (gray dashed line) at 1 Hz tone repetition rate (black), 2 Hz tone repetition rate (blue) and 4 Hz tone repetition rate (red). (1 Hz: *p* = 0.0019, *n* = 9 recordings from six mice. 2 Hz: *p* = 0.0079, *n* = 9 recordings from six mice. 4 Hz: *p* = 0.0006, *n* = 9 recordings from six mice. One-sample *t* tests). (F) Cartoon circuit diagram showing Jaws-expressing PV neurons (red) and GCaMP6f-expressing SOM neurons (green). (G) Average SOM neuron response amplitudes to the 1 Hz tone repetition rate repeated sound paradigm standard tones in both light-off control (black) and light-on PV neuron inactivation (yellow). Responses represented as light-on responses normalized to the corresponding light-off control first standard tone response: Response_n_/Control Response_1_. (1 Hz Light-off v. light-on: *p* = 7.102e−6, *n* = 7 recordings from four mice. Two-way repeated measures ANOVA with Tukey multiple comparisons). (H) Average SOM neuron response amplitudes to the 2 Hz tone repetition rate repeated sound paradigm standard tones in both light-off control (blue) and light-on PV neuron inactivation (yellow). Responses represented as light-on responses normalized to the corresponding light-off control first standard tone response: Response_n_/Control Response_1_. (2 Hz light-off v. light-on: *p* = 6.011e−5, *n* = 5 recordings from three mice. Two-way repeated measures ANOVA with Tukey multiple comparisons). (I) Average SOM neuron response amplitudes to the 4 Hz tone repetition rate repeated sound paradigm standard tones in both light-off control (red) and light-on PV neuron inactivation (yellow). Responses represented as light-on responses normalized to the corresponding light-off control first standard tone response: Response_n_/Control Response_1_. (4 Hz light-off v. light-on: *p* = 2.616e−7, *n* = 6 recordings from three mice. Two-way repeated measures ANOVA with Tukey multiple comparisons). (J) Bar plots showing the average change in SOM neuron calcium fluorescence response amplitudes during light-on PV neuron inactivation trials, normalized to the light-off trials, compared to no change (gray dashed line) at 1 Hz tone repetition rate (black), 2 Hz tone repetition rate (blue), and 4 Hz tone repetition rate (red). (1 Hz: *p* = 0.0099, *n* = 7 recordings from four mice. 2 Hz: *p* = 0.0036, *n* = 5 recordings from three mice. 4 Hz: *p* = 0.0013, *n* = 6 recordings from three mice. One-sample *t* tests). Asterisks indicate significant *p* values. Data are represented as mean ± SEM. See Table S1 for detailed statistics.

Our results suggest that corticocollicular neurons receive interneuron-type specific effects that combine to shape their stimulus-specific adaptation. Namely, SOM neurons function to enhance sound-evoked responses at lower tone repetition rates and PV neurons function to suppress sound-evoked responses at higher tone repetition rates. These complementary and opposing effects from these distinct populations of inhibitory neurons could arise from a purely disinhibitory hierarchy between SOM and PV neurons^44^ in which SOM neurons shape principal neuron responses via disinhibition of PV neurons.^33^ Alternatively, SOM and PV neurons could serve distinct but complementary functions, where each cell type contributes to the sound-evoked responses of corticocollicular neurons independently of the other cell type. To distinguish between these possibilities, we performed experiments to measure the effects of SOM neuron inactivation on PV neuron sound-evoked responses. We hypothesized that PV neurons would show disinhibitory responses to SOM neuron inactivation, but that this would not be sufficient to explain the enhancement of corticocollicular neuron sound-evoked responses by SOM neurons (Figure 2), thus supporting complementary roles of these interneurons during repetitive auditory stimulation. To perform this experiment, we induced the expression of GCaMP6f in PV neurons using an S5E2 enhancer AAV (STAR Methods, Figure S4).^45^^,^^46^ This AAV-based approach resulted in ∼94% of PV-positive neurons expressing GCaMP6f and only ∼6% of neurons that were not PV-positive expressing GCaMP6f in the auditory cortex from wildtype mice (Figure S4). We then injected this AAV along with a Cre-dependent Jaws AAV into the auditory cortex of SOM-Cre mice, which resulted in GCaMP6f expression in PV neurons and Jaws expression in SOM neurons in the same mice. We then performed experiments as above with interleaved light-off and light-on trials to assess the effects of SOM neuron inactivation on PV neuron sound-evoked responses. Consistent with our hypothesis, SOM neuron inactivation enhanced PV neuron responses to all tone repetition rates (Figures 4A–4E; Figures S5A and S5B). While these results are consistent with a disinhibition of PV neurons by SOM neurons, they also show that the effect of SOM neurons to enhance the sound-evoked responses of corticocollicular neurons is not simply carried out via PV neuron-mediated disinhibition of corticocollicular neurons.

To further confirm that disinhibition alone did not account for the enhancement of corticocollicular sound responses by SOM neurons, we performed experiments to measure the effects of PV neuron inactivation on SOM neuron sound-evoked responses. PV neurons could be acting as the primary controllers of disinhibition in this circuit, and the disinhibition of SOM neurons by PV neurons could drive the enhancement of corticocollicular neuron responses to sound. Alternatively, disinhibition could be present in both SOM neuron to PV neuron and PVneuron to SOM neuron circuits suggesting that these neurons are engaged in cooperative action to shape the context-dependent sound-evoked responses of corticocollicular neurons. To perform this experiment, we injected two AAVs into the auditory cortex of SOM-flp/PV-Cre double transgenic mice. We induced the expression of GCaMP6f in SOM neurons using a flp-dependent AAV (Method details) and the expression of Jaws in PV neurons with a Cre-dependent AAV. We then performed experiments as above with interleaved light-off and light-on trials to assess the effects of PV neuron inactivation on SOM neuron sound-evoked calcium responses. Consistent with our hypothesis, PV neuron inactivation enhanced SOM neuron responses to all tone repetition rates (Figures 4F–4J; Figures S5C and S5D). These results suggest parallel and complementary roles for SOM and PV neuron populations in shaping corticocollicular neuron responses to repetitive sound stimuli. While these results confirm functional disinhibitory networks between SOM neurons and PV neurons, the disinhibitory connections from SOM neurons to PV neurons, or from PV neurons to SOM neurons, are not sufficient to explain the observed SOM neuron-mediated enhancement of corticocollicular neuron responses to repetitive auditory stimuli. These results suggest that auditory cortical neurons operate in a continuum whereby SOM neuron enhancement and PV neuron suppression change as a function of the repetition rate of the sounds presented, suggesting that SOM and PV neurons cooperate to fine-tune stimulus-specific adaptation of corticocollicular neurons in the mouse auditory cortex, depending on the specific temporal structure of the sensory inputs. Together, our findings support the conclusions that (1) the effects of inhibition can be different for different types of neurons during cortical processing, (2) that SOM and PV neurons in the auditory cortex engage in multiple operating regimes that depend on the temporal structure of sensory inputs and sensory context, and (3) that SOM neurons can enhance the response of cortical principal neurons to sounds that occur at low repetition rates.

## Discussion

In this study we varied the rate at which repeated tones were presented to the mice and found that these different contexts (slow vs. fast tone repetition rates) were associated with distinct roles for SOM and PV neurons. Thus, we identify multiple operating regimes for SOM and PV neurons in the auditory cortex to provide complementary and opposing support for the stimulus-specific adaptation of corticocollicular neurons. This cooperative scheme exists in the presence of the direct disinhibitory pathways that have been previously identified for PV and SOM neurons in various cortical areas.^13^^,^^29^^,^^47^^,^^48^^,^^49^ Thus, our findings expand the roles of SOM and PV neurons to include a continuum of cooperative action that can operate to enhance and suppress the sound-evoked responses of cortical neurons. By combining multiple transgenic mouse lines, fluorescent calcium reporters, and optogenetic tools, our results suggest that SOM and PV neurons can operate in different inhibitory modes, depending on the context in which they are activated by sounds. Whether these combined inhibitory effects of SOM and PV neurons are specific to corticocollicular neurons, apply to other types of corticofugal neurons, or are a general feature of auditory cortical neurons remains an unanswered question. Previous studies on the role of SOM and PV neurons in auditory stimulus-specific adaptation have used faster tone repetition rates of 3–4 Hz and focused on principal neurons in superficial cortical layers.^14^^,^^27^ At these tone repetition rates, we observed that PV neurons acted to suppress sound-evoked responses and SOM neurons had no significant effects, but at lower tone repetition rates in the same animals, our results show that SOM and PV neurons have differential and opposing effects on corticocollicular neurons. Thus, the temporal structure of sensory inputs is a crucial parameter governing the recruitment of different populations of inhibitory interneurons in cortical circuits. Although not examined here, this range of tone repetition rates are consistent with ethological relevant rates of “syllables” observed in mouse con-specific vocalizations which can vary from two to over ten per second depending on the behavioral state of the animals.^50^^,^^51^^,^^52^ Together, these findings suggest that there are different operating regimes of interneurons that can be engaged by different temporal structures of stimulus inputs. These different regimes could also be engaged by changes in long-range neuromodulatory inputs from the basal forebrain, such as cholinergic inputs from the nucleus basalis that can shape SOM and PV neuron activity,^53^ that could also display sound context-dependent activity. Future experiments examining the specific roles for SOM and PV neurons on specific classes of principal neurons, and on the recurrent circuits that these neurons form with each other^54^^,^^55^^,^^56^^,^^57^ will be required to generalize these findings to the larger context of cortical circuit function.

Our results expand the functions of interneurons in cortical computations. Previous studies have reported that SOM and PV neurons can have divisive (non-linear scaling) or subtractive (linear scaling) effects on sensory processing,^58^^,^^59^ which can change based on the baseline activity of the neurons.^60^ Our results suggest that recent stimulus experience can also change the effects of interneurons, potentially by altering the activity rates in a stimulus-specific manner. In addition, our results were obtained using louder sounds (80 dB SPL vs. 65 or 70 dB SPL^14^^,^^27^), which could also be an important feature in setting the operating regime of SOM and PV neurons that we observed. Our results also support the view of an inhibition-stabilized operating regime of cortical circuits, where disinhibitory connections are balanced by the resulting increased recruitment of inhibition that occurs during sensory processing.^29^^,^^30^^,^^61^^,^^62^^,^^63^ Together, our results offer a physiologically constrained set of parameters that can be incorporated into circuit models of sensory adaptation by cortical neurons. Future experiments directly measuring the effects of temporal structure, stimulus intensity, and neuronal identity will be required to more fully characterize how the different operating regimes of excitation and inhibition serve to support the stimulus-dependent responses of cortical neurons.

There is a growing appreciation for the diversity of SOM neurons, a large family of interneurons that comprise up to 13 subtypes^64^ which can be identified by differences in their molecular and genetic signature,^28^^,^^65^^,^^66^^,^^67^ their morphology,^68^ and their electrophysiological characteristics.^67^ In this study, we find opposing and complementary effects of SOM neurons on the adaptation of different types of cortical neurons. One explanation for these findings could be that distinct subtypes of SOM neurons provide input to PV neurons and corticocollicular neurons. Thus, the effects of SOM neurons to suppress the sound-evoked responses of PV neurons and enhance the sound-evoked responses of corticocollicular neurons could arise because different subtypes of SOM neurons preferentially engage in different cortical circuits with PV neurons vs. corticocollicular neurons during sensory processing. In addition, possible differences in the expression levels and efficacy of inactivating opsins between SOM neuron subtypes and compared to PV neurons may exist, which could also contribute to the differential effects we observed. Future experiments using more restricted approaches to express opsins and/or calcium sensors in defined subtypes of SOM neurons will be required to address this issue. Future experiments using complementary approaches to neuronal inactivation, such as light-activated hyperpolarizing proton pumps (e.g., archaerhodopsins)^69^ other types of chloride pumps (e.g., halorhodopsins^70^), and/or chemogenetic approaches using designer receptors exclusively activated by designer drugs (DREADDS^71^), could be used to directly address these possibilities.

In this study, we observed that the effects of PV neuron inactivation were less dramatic than the effects of SOM neuron inactivation on corticocollicular neuron sound-evoked responses, which may suggest that the effects of PV neurons could be weaker and/or more variable than the effects of SOM neurons on corticocollicular neurons. Because the effects of PV neuron inactivation to enhance sound-evoked responses were more robust for SOM neurons (Figure 4) compared to corticocollicular neurons (Figure 3), this supports the notion that PV neurons can exert different effects on different types of neurons in the auditory cortex. In addition, because PV neurons demonstrate stimulus-intensity-dependent recruitment,^42^^,^^72^^,^^73^ their inhibitory effects onto corticocollicular neurons may also be stimulus-intensity-dependent. This suggests that the relatively weak effects of PV neuron inactivation on corticocollicular neuron sound-evoked responses here may be related to the specific sound paradigms used in this study. Future experiments exploring a wider range of sound parameters on corticocollicular neuron responses will be required to address this question.

Together, our results expand the range of effects that PV and SOM neurons can have during stimulus-specific adaptation. Previous works on the effects of PV and SOM neuron inactivation in cortical layer 2/3 principal neurons have shown a variety of effects.^14^^,^^20^^,^^27^ Our results are consistent with this range of effects and suggest that different features of the stimuli used (e.g., intensity and repetition rate) can engage different functional aspects of PV and SOM neuron activity. Together with these previous studies, our results suggest that the specific combination of cell types, cortical layer, and stimulation parameters determine how the effects of PV and SOM neurons manifest during cortical processing. This supports the conclusion that these interneurons engage in different operating regimes that exist in a continuum depending on the context of how they are recruited. Future experiments to systematically vary different aspects of auditory inputs will be necessary to uncover fundamental principles that govern the effects of PV and SOM neurons on auditory processing.

### Limitations of the study

In this study, we used widefield calcium imaging combined with optogenetic manipulations to measure the neuronal responses to sounds. These widefield signals represent the calcium transients in multiple neurons at once and likely arise from neuronal dendrites in addition to neuronal somata.^35^ Previous work has shown good accordance between the widefield calcium responses of PV, SOM, and corticocollicular neurons and the underlying somatic responses of these cell types,^35^^,^^42^^,^^74^^,^^75^ but this was not directly measured in this study, therefore future work to understand stimulus-specific adaptation at the level of individual neuronal somata will be required to address this gap. In addition, this study is based on calcium responses measured with GCaMP6f, jGCaMP7b, and jGCaMP8m. Since different genetically encoded calcium sensors have different dynamic ranges and calcium sensitivity^76^^,^^77^^,^^78^^,^^79^ the relationship between calcium transients and action potential firing in different cell types cannot be easily inferred. Future work using multiple calcium sensors or using other approaches such as electrical recording or voltage-sensitive indicators will be required to more reliably link the fluorescent responses of specific neurons to their underlying activity rates. Additionally, this study did not explore if different viral vectors are preferentially expressed by SOM neurons vs. PV neurons and so future experiments directly assessing this possibility will be required to address this question. We have limited our optogenetic manipulations to cortical neurons, and so potential contributions of long-range projections, e.g., from the basal forebrain,^80^ to the adaptation of corticocollicular neurons were not explored. We have also limited our optogenetic manipulations to either SOM or PV neurons, and have not explored the effects of inactivating both SOM and PV neurons together, so future experiments linking manipulations of multiple classes of interneurons to the sound-evoked responses of principal neurons will be required to explore these effects. This study is based on data collected from both male and female mice, but we did not explore potential sex differences in the effects of SOM and PV neurons on the sound-evoked responses of corticocollicular neurons. In this study, we did not assess the potential for the opsin Jaws to have different effects on SOM vs. PV neuron activity and so future studies understanding the effects of opsins in these potentially diverse populations of neurons will be required to address this issue. In this study, we also did not explore the effects of optogenetic manipulations of other classes of inhibitory neurons such as vasoactive intestinal peptide (VIP) neurons, which can have diverse inhibitory and dis-inhibitory effects on both SOM and PV neurons,^47^^,^^81^ and so future studies about the contribution of VIP neurons to the stimulus-specific adaptation of SOM, PV, and corticocollicular neurons will be required to understand this issue.

## Resource availability

### Lead contact

Further information and requests for resources and reagents should be directed to and will be fulfilled by the lead contact, Charles T. Anderson (charles.anderson@hsc.wvu.edu).

### Materials availability

This study did not generate new unique reagents.

### Data and code availability

• All data generated in this study are available in the article.
• This study does not report original code.
• Any additional information required to reanalyze the data reported in this study is available from the lead contact upon request.

## Acknowledgments

This work was supported by the 10.13039/100000057National Institute of General Medical Sciences: R35-GM138023 (C.T.A.) and T32-GM133369 (R.R.); and the 10.13039/100000049National Institute on Aging: T32-AG052375 (K.B.). This material is based upon work supported by the 10.13039/100000001National Science Foundation under grant 2326758 (C.T.A.). This material is based upon work supported by the 10.13039/100000001National Science Foundation under cooperative agreement no. OIA-2242771 (C.T.A.). We thank the West Virginia University Microscope Imaging Facility, which has been supported by the 10.13039/100018239WVU Cancer Institute and 10.13039/100000002NIH grants P20RR016440, P30GM103488, U54GM104942, and P20GM103434.

## Author contributions

Conceptualization, P.T.R.B., M.M., and C.T.A.; methodology, P.T.R.B., M.M., and C.T.A.; investigation, P.T.R.B., M.M., E.T., K.B., R.R., and H.L.; visualization, P.T.R.B., M.M., E.T., and C.T.A.; supervision, C.T.A.; writing – original draft, P.T.R.B. and C.T.A.; writing – review and editing, P.T.R.B., E.T., K.B., R.R., and C.T.A.

## Declaration of interests

The authors declare that they have no competing interests.

## STAR★Methods

### Key resources table

**Table:** 

REAGENT or RESOURCE	SOURCE	IDENTIFIER
**Viral Vectors**		

pGP-AAV-*syn*-FLEX-jGCaMP7b-WPRE	Addgene	Cat#: 104493
pGP-AAV-*syn*-jGCaMP8m-WPRE (retrograde)	Addgene	Cat#: 162375
pAAV-S5E2-GCaMP6f	Addgene	Cat#: 135632
pAAV-Ef1a-Coff/Fon- GCaMP6f	Addgene	Cat#: 137124
pAAV-CAG-FLEX-rc [Jaws-KGC-GFP-ER2]	Addgene	Cat#: 84445

**Experimental models: Organisms/strains**		

Mouse: SOM-IRES-Cre	The Jackson Laboratory	RRID: IMSR_JAX:013044
Mouse: B6 Pvalb-IRES-Cre	The Jackson Laboratory	RRID: IMSR_JAX:017320
Mouse: SOM-IRES-FLPo (C57BL/6J)	The Jackson Laboratory	RRID: IMSR_JAX:031629

**Software and algorithms**		

ImageJ	FIJI	RRID:SCR_003070
MATLAB	Mathworks	RRID:SCR_001622
ephus	Suter et al.^82^	Suter et al.^82^
Wavesurfer	Janelia Farms	RRID:SCR_021529
GraphPad Prism 9	GraphPad	RRID:SCR_000306

**Reagents for electrophysiological experiments**		

Sodium Chloride	Sigma-Aldrich	cat# S9888
Choline chloride	Sigma Aldrich	cat# C7017
NaHCO_3_	Sigma-Aldrich	cat# S6014
D-Glucose	Sigma-Aldrich	cat# G8270
sodium ascorbate	Sigma-Aldrich	cat# A7631
sodium pyruvate	Sigma Aldrich	cat# P8574
Potassium Chloride	Sigma-Aldrich	cat# P9541
Calcium Chloride	Sigma-Aldrich	cat# 499609
Magnesium Chloride	Sigma-Aldrich	cat# 255777
HEPES	Sigma-Aldrich	cat# 54457
potassium-gluconate	Sigma-Aldrich	cat# P1847
Na_2_-ATP	Sigma-Aldrich	cat# A26209
Tris-GTP	Sigma-Aldrich	cat# G9002
Tris-phosphocreatine	Sigma-Aldrich	cat# P1937
EGTA	Sigma-Aldrich	cat# 324626
Na-ascorbate	Sigma-Aldrich	cat# A4034

**Antibodies**		

Parvalbumin antibody	rabbit host, Abcam	ab11421
Somatostatin antibody	rabbit host, Abcam	ab111912
Goat anti-rabbit Alexa Fluor 594	Jackson Immunoresearch	111-585-144

### Experimental model and study participant details

Male and female wild type C57Bl/6 (Jackson Laboratories), SOM-Cre (Strain: 013044, Jackson Laboratories), SOM-flp (Strain: 031629, Jackson Laboratories) and PV-Cre (Strain: 017320, Jackson Laboratories) mice aged postnatal day (P) 21-P56 were used in this study. We did not explore potential sex differences in the results reported here. All procedures were approved by the West Virginia University Institutional Animal Care and Use Committee (IACUC); IACUC approval number 1708008945. Mice were maintained on a regular light cycle (12 h light: 12 h dark) with constant access to food, water, and nesting material for environmental enrichment. Single housed mice were provided with additional enrichment.

### Method details

#### Stereotaxic surgery

Surgical procedures are similar to those previously described.^35^ Here, mice aged P21 through P35 were anesthetized with inhaled isoflurane (Induction: 3% in oxygen, maintenance 1.5% in oxygen) and secured in a stereotaxic frame (Stoelting). Core body temperature was maintained at ∼37°C with a feedback controlled heating pad (Stoelting). Eyes were protected with ophthalmic ointment during the procedure. Lidocaine (1%) was injected under the scalp, and an incision was made into the skin at the midline to expose the skull. Using a 27-gauge needle as a scalpel, small craniotomies (∼0.4 mm diameter) were made over the inferior colliculus (coordinates 1.3 mm posterior and 1.0 mm lateral to lambda), and/or the auditory cortex (coordinates 0.0 mm posterior, 3.7 mm lateral to lambda). Borosilicate glass pipettes (VWR) were pulled to a shallow taper (length > 1 cm, tip diameter ∼30 μm), and were advanced into the region of interest at an angle of ∼25° off the horizontal plane. Injection pipettes were backfilled with mineral oil (Sigma) and filled with retro-AAV-hSyn1-jGCaMP8m (for inferior colliculus injections, Figures 1, 2, and 3), AAV9-hSyn1-FLEX-Jaws (Figures 2, 3, and 4), AAV9-hSyn1-FLEX-GFP (Figure S2), AAV9-hSyn1-FLEX-jGCaMP7b (Figure S2), pAAV-Ef1a-fDIO-GCaMP6f (Figures 4F–4J and S4) or pAAV-S5E2-GCaMP6f (Figures 4A–4E and S5; titer 5e^12^ – 5e^13^ genome copies/mL, Addgene). They were connected to 5 μL glass syringes (Hamilton) via capillary tubing and controlled with syringe pumps (World Precision Instruments). Pipettes were inserted 1.5 mm deep (inferior colliculus) or 0.55 mm deep (auditory cortex) into the craniotomy and 0.9 μL of adeno-associated virus (AAV) was injected at 0.3 μL per minute for 3 min. After injections, the pipettes were left in place for 2 min prior to removal and then the scalp of the mouse was closed with cyanoacrylate adhesive. Mice received an injection of the non-steroidal anti-inflammatory drug meloxicam (4 mg per kg) during the procedure and a diet of meloxicam tablets (Bio-Serv) for 72 h after surgery. Mice were monitored for postoperative stress and pain. Experiments were performed 7–10 days (flex AAV) or 14–21 days (retrograde AAV) post-injection to ensure delivery of the viral payload.

#### Widefield calcium imaging

Imaging procedures are similar to those previously described.^35^ Here, 7–10 days (flex AAV) or 14–21 days (retrograde AAV) after AAV injections, mice were prepared for *in vivo* calcium imaging. Mice were anesthetized with inhaled isoflurane (induction: 3% in oxygen, maintenance: 1.5% in oxygen) and positioned into a custom head holder. Core body temperature was maintained at ∼37 °C with a heating pad and eyes were protected with ophthalmic ointment. Lidocaine (1%) was injected under the scalp and an incision (∼1.5 cm long) was made into the skin over the right temporal cortex. The head of the mouse was rotated ∼45° in the coronal plane to align the pial surface of the right temporal cortex with the imaging plane of the upright microscope optics. The skull of the mouse was secured to the head holder using dental acrylic (Stoelting) and cyanoacrylate adhesive, and the mouse received an injection of chlorprothixene (1.5 mg/kg intraperitoneal). In all experiments, recordings were obtained from mice injected with chlorprothixene: a sedative that shows antagonism for subsets of histamine,^83^ serotonin,^84^^,^^85^ dopamine,^86^ and muscarinic^84^ receptors. We did not assess the effects of chlorprothixene on the sound-evoked responses of neurons, but it was consistently used in all *in vivo* imaging experiments in this study. A dental acrylic reservoir was created to hold warm artificial cerebrospinal fluid (ACSF) over the exposed skull. While under anesthesia, we performed a craniotomy (∼1 mm^2^) of the skull over the temporal cortex and then mice were then positioned under the microscope objective in a sound and light attenuation chamber containing the microscope and a calibrated speaker (ES1, Tucker-Davis). We removed the isoflurane from the oxygen flowing to the animal and then presented auditory stimuli. Sounds were delivered from a free-field speaker ∼10 cm from the left ear of the animal (MF1 speaker, SA1 driver, Tucker-Davis Technologies), controlled by a digital to analog converter using an output rate of 250 kHz (USB-6343, National Instruments); sound stimulus intensity was calibrated with ¼ inch microphone (Brüel & Kjær). We used ephus^82^ and Wavesurfer 1.0.6 (Janelia Farms) to generate the calibrated sound waveforms and synchronize the sound delivery and image acquisition hardware. To locate A1, we presented a 500 msec, 80 dB SPL, 8 kHz tone with 5 msec cosine ramp to the animal while illuminating the cortex with a blue LED (M490L2, Thorlabs). Although previous studies have reported escape behaviors elicited by these sound intensities,^87^ we did not observe mouse movements in response to these sounds. We imaged the change in green GCaMP emission with epifluorescence optics (eGFP filter set, U-N41017, Olympus) and a 4× objective (Olympus) using a cooled CCD camera (Rolera, Q-Imaging). Images were acquired at frame rate of 20 Hz and resolution of 200 x 150 pixels (using 8X spatial binning), covering a field of view of 3 mm × 2.2 mm. To locate A1 we normalized the sound-evoked change in fluorescence after sound presentation (ΔF) to the baseline fluorescence (F), where F is the average fluorescence of 1 s preceding the sound onset (for each pixel in the movie). We applied a two-dimensional, low-pass Butterworth filter to each frame of the ΔF/F movie and then created an image consisting of a temporal average of 10 consecutive frames (0.5 s) beginning at the end of the sound stimulus. This image indicated two sound-responsive regions corresponding to the low-frequency tonotopic areas of A1 and the AAF.^88^^,^^89^^,^^90^

After locating A1 we performed widefield imaging of GCaMP-expression. We presented a repeated sound paradigm auditory stimulus, with ten standard tones followed by a deviant tone, using both an 8 kHz standard tone with a 16 kHz deviant tone, and a 16 kHz standard tone with an 8 kHz deviant tone, with tone durations of 100 msec with 5 msec cosine ramps. The sounds were presented at three tone repetition rates: 1 Hz (1 s inter-stimulus interval), 2 Hz (0.5 s inter-stimulus interval), and 4 Hz (0.25 s inter-stimulus interval). During light-on trials, orange light from an LED (Thorlabs M625F2), was delivered by a 400 μm core fiberoptic cable (Thorlabs M118L02, irradiance ∼4 mW/cm^2^ at the cortical surface) positioned over the identified A1, and used to optically inactivate Jaws-expressing neurons during the sound presentation. Each animal received 5–10 presentations of each stimulus trial in both light-off and light-on conditions. Light-off and light-on trials were interleaved. The time between trials (each trial contained ten standard tones and one deviant tone) was at least 30 s to allow recovery from adaptation during the previous trial. The sequence of tone repetition rate and standard tone frequency blocks presented were randomized during each experiment to account for any potential time-related changes in fluorescence response amplitudes. To reduce the contribution of any potential locomotion-related changes in calcium fluorescence, movies with obvious motion artifacts were excluded from further processing. To quantify the widefield calcium fluorescence response to sound, we identified an ROI from within A1 that responded to the sound presentation. These ROIs were obtained from the surface of the brain and we did not further parse to what extent these signals represented single neurons, clusters of neurons, or neuronal dendrites. The pixels in each ROI were averaged and converted into ΔF/F as above. We then averaged the fluorescent response for 5–10 presentations of the same sound trail for each ROI. Response amplitudes were measured as peak-trough of the fluorescence response to each tone and normalized either to the first control (light-off) sound response in a train (Response_n_/Control_1_) or to the corresponding control (light-off) response (Response_n_/Control_n_).

#### Perfusions

Perfusions were performed according to previously described procedures.^91^ Here, male and female mice which had undergone stereotaxic injections of pAAV-S5E2-GCaMP6f or AAV9-hSyn1-FLEX-jGCaMP7b into the auditory cortex 7–10 days prior were anesthetized and perfused transcardially using carbogenated artificial cerebral spinal fluid (ACSF) containing the following (in mM): 130 NaCl, 3 KCl, 2.4 CaCl_2_, 1.3 MgCl_2_, 20 NaHCO_3_, 3 HEPES, and 10 D-glucose (saturated with 95% O_2_/5% CO_2_ (vol/vol), ∼300mOsm, pH 7.25–7.35), followed by 4% paraformaldehyde (PFA) with 0.0028% glutaraldehyde in phosphate buffered saline (PBS). Brains were immediately removed and placed into PFA overnight. Brains were then cryopreserved in 30% sucrose in PBS and sectioned coronally at 25 μm on a microtome (Thermo Fisher Scientific Sliding microtome Microm HM450) with a BFS-40MPA freezing stage (Physitemp). Sections were stored free-floating in 0.01% sodium azide in PBS at 4°C.

#### Immunohistochemistry

Immunohistochemical procedures were performed according to previously described procedures.^91^ Here, sections from the auditory cortex were first blocked for 1 h at room temperature in a blocking buffer (1% bovine serum albumin, 10% fetal bovine serum, 1% Triton X-100 in PBS). Sections were then incubated for 48 h with primary antibody (parvalbumin antibody, rabbit host, Abcam: ab11421; or somatostatin antibody, rabbit host, Abcam: ab111912) in a blocking buffer and washed three times for 10 min with PBS. Sections were incubated with secondary antibody (Goat anti-rabbit Alexa Fluor 594, Jackson Immunoresearch, 111-585-144) in a blocking buffer (1:500) for 2 h at room temperature, washed three times in PBS for 10 min, washed two times in 0.1 M phosphate buffer for 5 min, and mounted to glass slices with ProLong Glass Antifade Mountant (Invitrogen) and covered with a No. 1.5 glass coverslip.

#### Fluorescent imaging & analysis

Fixed and labeled brain slices of auditory cortex were imaged using an Olympus MVX MacroView microscope with a Hamamatsu ORCA_Flash4.0 v2 sCMOS camera (C11440-22CU) for data presented in Figure S4 or with a confocal microscope (Zeiss LSM 710 confocal with a 20x Plan Apochromat/0.8 NA DIC objective) for data presented in Figure S2. Image ROIs were selected in the auditory cortex based on the presence of GCaMP6f or jGCaMP7b expression from the viral injections. In each ROI, GCaMP6f-positive or jGCaMP7b-positive cells were identified. From these GCaMP-positive cells, the number that were also parvalbumin (PV)-positive or somatostatin (SST)-positive cells was counted. The overlap between GCaMP6f and PV or jGCaMP7b and SST is reported as a percent of the total number of GCaMP6f- or jGCaMP7b-positive cells.

#### Electrophysiology recordings

Acute brain slice experiments are similar to those that have been previously described,^92^ and here, were performed 7–10 days post injection with pAAV-S5E2-GCaMP6f. Slices were examined during experiments to confirm accurate placement of the injection sites. Brain slices of auditory cortex were cut in chilled carbogenated choline-based solution of the following composition (in mM): 110 Choline chloride, 25 NaHCO_3_, 25 D-Glucose, 11.6 sodium ascorbate, 3.1 sodium pyruvate, 2.5 KCl, 0.5 CaCl_2_, 7 MgCl_2_. Brain slice electrophysiology experiments were carried out using carbogenated artificial cerebrospinal fluid (ACSF) with the following composition (in mM): 130 NaCl, 3 KCl, 2.4 CaCl_2_, 1.3 MgCl_2_, 20 NaHCO_3_, 3 HEPES, 10 D-glucose, saturated with 95% O_2_/5% CO_2_ (vol/vol), pH 7.25–7.35, ∼300 mOsm. Mice were first anesthetized with isoflurane and then immediately decapitated. Brains were rapidly removed and coronal slices (300 μm) of the cortex were prepared in chilled choline chloride cutting solution using a vibratome (VT1200 S; Leica). Slices were then transferred into a holding chamber of carbogenated ACSF and incubated for ∼30 min at 35 °C and then incubated at room temperature for ∼30 min before electrophysiological experiments were performed. For electrophysiological experiments, slices were transferred into the recording chamber and perfused with carbogenated ACSF at a rate of 1–2 mL/min. Recordings were performed at 30°C–32 °C using an in-line heating system (Warner Instruments). Electrophysiological recordings were made using an amplifier (MultiClamp-700B, Axon Instruments), a digital to analog converter (USB-6229, National Instruments), and ephus.^82^ Current-clamp recordings were conducted using borosilicate pipettes (Warner Instruments) pulled to tip resistances of 3–5 MΩ (Sutter Instruments) filled with a potassium-based internal solution with the following composition (in mM): 128 potassium-gluconate, 10 HEPES, 4 MgCl_2_, 4 Na_2_-ATP, 0.3 Tris-GTP, 10 Tris-phosphocreatine, 1 potassium-EGTA, 3 Na-ascorbate. (pH = 7.23, 303 mOsm). Data were sampled at 10 kHz and lowpass filtered at 2–4 kHz. Analysis was performed using MATLAB. Cells were held in current clamp configuration and 1 s current steps of −50 pA, 0 pA, and +250 pA were applied. Fluorescence changes during these current steps were recorded with epifluorescence optics (eGFP filter set, U-N41017, Olympus) and a 4× objective (Olympus) using a cooled CCD camera (Rolera, Q-Imaging) at ∼20 Hz frame rate.

### Quantification and statistical analysis

For every comparison in the manuscript we describe the statistical test used, the exact value of n, what n represents, the measure of central tendency (e.g., median or mean), and the definition of the error bars in the corresponding figure legend and in Table S1. Analysis was performed with MATLAB (Mathworks), Fiji,^93^ and Prism (GraphPad). For statistical comparisons in Figure 1F (involving 2 and 4 Hz groups), Figure 1G (involving 1 and 2 Hz groups), Figures 2G and 3G, Figures 4E, 4J, S1B, S1C, S2F, S2H, S3B, S3C, S5B, and S5D, Student paired t-tests and one-sample t-tests were used because the group data passed Lilliefors test for normality. In Figure S2H, Student t-tests were used because the group data passed Lilliefors test for normality. For statistical comparisons in Figures 2D–2F, 3D–3F, 4B, 4C, 4D, 4G–4I, 2-way repeated measures ANOVA with Greenhouse-Geisser correction for sphericity was used. For statistical comparisons in Figure 1F (involving the 1 Hz group), Figure 1G (involving the 4Hz group), and Figure 3G (involving the 1 Hz group), Wilcoxon signed-rank tests were used because the group data did not pass the Lilliefors test for normality. The Lilliefors test was calculated using the MATLAB function lillietest. Significance levels were determined based on Holm-Bonferroni post hoc correction for multiple comparisons. Statistically significant differences are denoted in figures with an asterisk (∗). Error bars represent ±SEM.

## Supplemental information

Document S1. Figures S1−S5

Table S1. Detailed statistical appendix for all figures
