# Supplementary material for: Context-dependent effects of SOM neurons to enhance the responses of corticocollicular neurons to repetitive sounds
Source: iScience. 2026 Mar 30;29(5):115536. doi: 10.1016/j.isci.2026.115536 (PMC13122691; doi:10.1016/j.isci.2026.115536)
Supplement: Document S1. Figures S1−S5 [file mmc1.pdf]

iScience, Volume *29*

Supplemental information

Context-dependent effects of SOM neurons to enhance the responses of corticocollicular neurons to repetitive sounds

Philip T.R. Bender, Mason McCollum, Emma Trate, Kaitlin Bainer, Rayli Ruby, Hui Li, and Charles T. Anderson

iScience, Volume ■■

Supplemental information

Context-dependent effects of SOM neurons to enhance the responses of corticocollicular neurons to repetitive sounds

Philip T.R. Bender, Mason McCollum, Emma Trate, Kaitlin Bainer, Rayli Ruby, Hui Li, and Charles T. Anderson

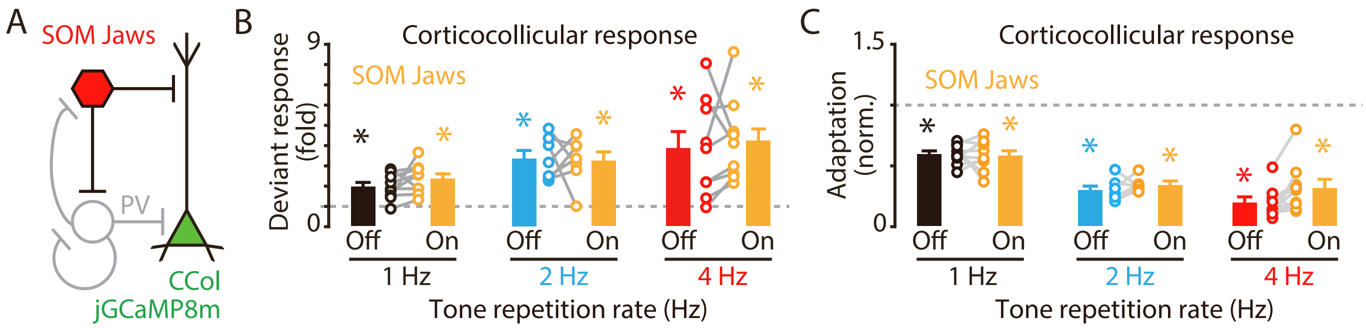

Fig. S1: Corticocollicular neuron deviance detection during inactivation of SOM neurons by Jaws

(A) Cartoon circuit diagram showing Jaws-expressing SOM neurons (red) and jGCaMP8m-expressing corticocollicular neurons (CCol, green).

(B) Responses of corticocollicular neurons to a deviant tone after a train of ten standard tones at different tone repetition rates. Average difference between the fluorescence response amplitude to the tenth (last) standard tone (grey dashed line) and the fluorescence response amplitude to the deviant tone in the stimulus train in light-off controls and light-on inactivation of SOM neurons at 1 Hz (light-off: black, light-on: yellow), 2 Hz (light-off: blue, light-on: yellow), and 4 Hz (light-off: red, light-on: yellow) tone repetition rates (1 Hz; light-off: $p = 0.0032$, light-on: $p = 8.305e-4$, light-off v. light-on: $p = 0.1998$, $n = 9$ recordings from 5 mice. Paired t-test. 2 Hz; light-off: $p = 0.0012$, light-on: $p = 0.0026$, light-off v. light-on: $p = 0.8703$, $n = 7$ recordings from 4 mice. Paired t-test. 4 Hz; light-off: $p = 0.009$, light-on: $p = 0.0018$, light-off v. light-on: $p = 0.8169$, $n = 9$ recordings from 5 mice. Paired t-test.)

(C) Adaptation of corticocollicular neurons to repeated standard tones at different tone repetition rates. Average difference between the fluorescence response amplitude to the first tone (grey dashed line) and the fluorescence response amplitude to the tenth (last) standard tone in the stimulus train in light-off controls and light-on inactivation of SOM neurons at 1 Hz (light-off: black, light-on: yellow), 2 Hz (light-off: blue, light-on: yellow), and 4 Hz (light-off: red, light-on: yellow) tone repetition rates (1 Hz light off: $p = 2.20e-07$, light-on: $p = 8.51e-06$, light-off v. light-on $p = 0.0216$, $n = 9$ recordings from 5 mice. 2 Hz light off: $p = 1.28e-07$, light-on: $p = 5.01e-08$, light-off v. light-on $p = 0.0137$, $n = 7$ recordings from 4 mice. 4 Hz light off: $p = 7.49e-08$, light-on: $p = 4.48e-10$, light-off v. light-on $p = 0.2801$, $n = 9$ recordings from 5 mice. Paired t-test.) Asterisks indicate significant p values. Data are represented as mean ± SEM. See Table S1 for detailed statistics.

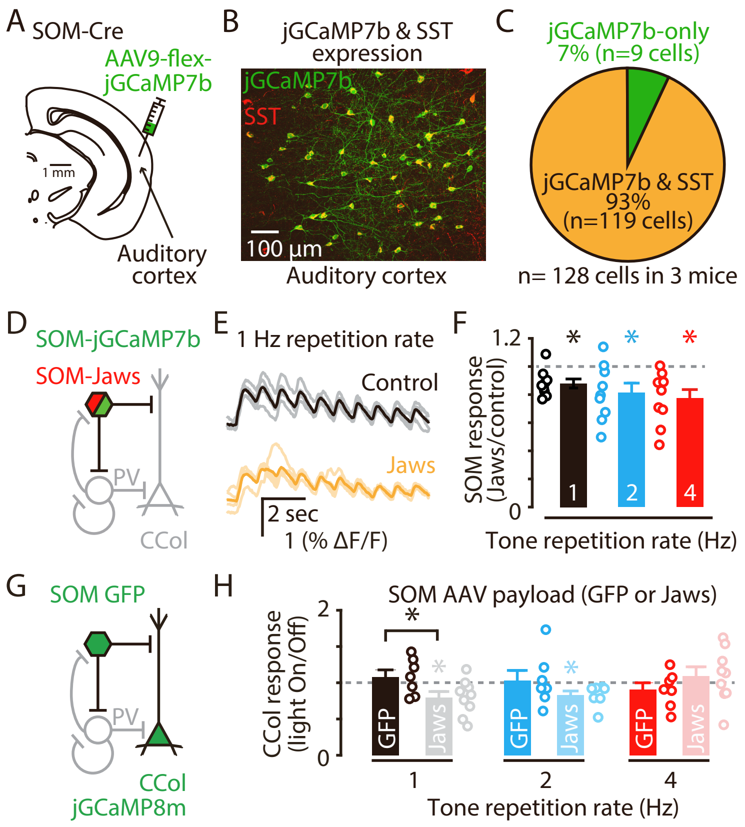

Fig. S2: Jaws activation inhibits SOM neuron responses to trains of sounds and Jaws is required for light to modulate corticocollicular neuron responses to trains of sounds

(A) Cartoon showing the stereotaxic injection scheme for expressing the genetically encoded fluorescent calcium sensor jGCaMP7b in SOM-Cre mice.

(B) Example brain section in auditory cortex showing jGCaMP7b and somatostatin (SST) expression.

(C) Pie chart showing that ~93 % of cells that were jGCaMP7b-positive were also SST-positive (red and green) and ~7 % of cells were jGCaMP7b-positive but not SST-positive (green).

(D) Cartoon circuit diagram showing Jaws (red) and GCaMP7b (green)-expressing SOM neurons.

(E) Example traces showing the individual (lighter lines) and average GCaMP7b-mediated calcium fluorescence responses (darker lines) to the repeated sound paradigm at 1 Hz tone repetition rate in both light-off control (black) and light-on SOM neuron inactivation by Jaws (yellow). Deviant tone response is highlighted with an arrow.

(F) Bar plots showing the average change in SOM neuron calcium fluorescence response amplitudes during light-on trials, normalized to the light-off trials, compared to no change (grey dashed line) at 1 Hz tone repetition rate (black), 2 Hz tone repetition rate (blue) and 4 Hz tone repetition rate (red). (1 Hz: $p = 0.0052$. 2 Hz: $p = 0.0242$. 4 Hz: $p = 0.0067$; one-sample t-tests. 1 Hz v. 2 Hz: $p = 0.3774$. 1 Hz v. 4 Hz: $p = 0.1514$. 2 Hz v. 4 Hz: $p = 0.6252$, n = 9 recordings from 5 mice. Paired t-tests.)

(G) Cartoon circuit diagram showing GFP-expressing SOM neurons (green) and jGCaMP8m-expressing corticocollicular neurons (CCol, green).

(H) Bar plots showing the average change in corticocollicular neuron calcium fluorescence response amplitudes during light-on trials, normalized to the light-off trials, compared to no change (grey dashed line) between GFP AAV payload (darker bars) and Jaws AAV payload (lighter bars) at 1 Hz tone repetition rate (black), 2 Hz tone repetition rate (blue) and 4 Hz tone repetition rate (red). (GFP: 1 Hz: $p = 0.4967$. 2 Hz: $p = 0.8397$. 4 Hz: $p = 0.4336$. 1 Hz v. 2 Hz: $p = 0.7447$; one-sample t-tests. 1 Hz v. 4 Hz: $p = 0.3690$. 2 Hz v. 4 Hz: $p = 0.6125$; paired t-tests. 1 Hz GFP vs 1 Hz Jaws: $p = 0.0387$. 2 Hz GFP vs 2hz Jaws: $p = 0.1906$. 4 Hz GFP vs 4 Hz Jaws: $p = 0.266$ n = 6 recordings from 3 mice; t-tests.)

Asterisks indicate significant p values. Data are represented as mean ± SEM. See Table S1 for detailed statistics.

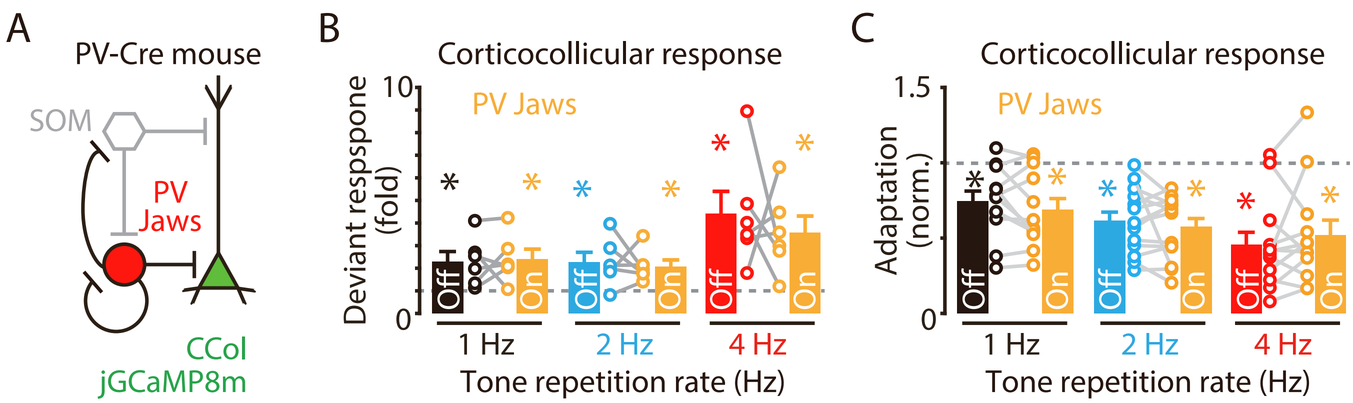

Fig. S3: Corticocollicular neuron deviance detection during inactivation of PV neurons by Jaws

(A) Cartoon circuit diagram showing Jaws-expressing PV neurons (red) and jGCaMP8m-expressing corticocollicular neurons (CCol, green).

(B) Responses of corticocollicular neurons to a deviant tone after a train of ten standard tones at different tone repetition rates. Average difference between the fluorescence response amplitude of the tenth (last) standard tone (grey dashed line) and the fluorescence response amplitude to the deviant tone in the stimulus train in light-off controls and light-on inactivation of PV neurons at 1 Hz (light-off: black, light-on: yellow), 2 Hz (light-off: blue, light-on: yellow), and 4 Hz (light-off: red, light-on: yellow) tone repetition rates (1 Hz; light-off: p = 0.0171, light-on: p = 0.0030; one-sample t-tests, light-off v. light-on: p = 0.8158; paired t-test, n = 12 recordings from 7 mice. Paired t-test. 2 Hz; light-off: p = 0.0068, light-on: p = 0.0018; one-sample t-tests, light-off v. light-on: p = 0.7673, n = 14 recordings from 8 mice; paired t-test. 4 Hz; light-off: p = 0.0071, light-on: p = 0.0007; one-sample t-tests, light-off v. light-on: p = 0.1581, n = 12 recordings from 7 mice; paired t-test.)

(C) Adaptation of corticocollicular neurons to repeated standard tones at different tone repetition rates. Average difference between the fluorescence response amplitude of the first tone (grey dashed line) and the fluorescence response amplitude to the tenth (last) standard tone in the stimulus train in light-off controls and light-on inactivation of PV neurons at 1 Hz (light-off: black, light-on: yellow), 2 Hz (light-off: blue, light-on: yellow), and 4 Hz (light-off: red, light-on: yellow) tone repetition rates (1 Hz light off: p = 0.0031, light-on: p = 0.0364; one-sample t-tests, light-off v. light-on p = 0.8133, n = 12 recordings from 7 mice; paired t-test. 2 Hz light off: p = 6.20e-06, light-on: p = 0.0026; one-sample t-tests, light-off v. light-on p = 0.0969, n = 14 recordings from 8 mice; paired t-test. 4 Hz light off: p = 9.98e-05, light-on: p = 0.0028; one-sample t-tests, light-off v. light-on p = 0.0739 n = 12 recordings from 7 mice; paired t-test.)

Asterisks indicate significant p values. Data are represented as mean ± SEM. See Table S1 for detailed statistics.

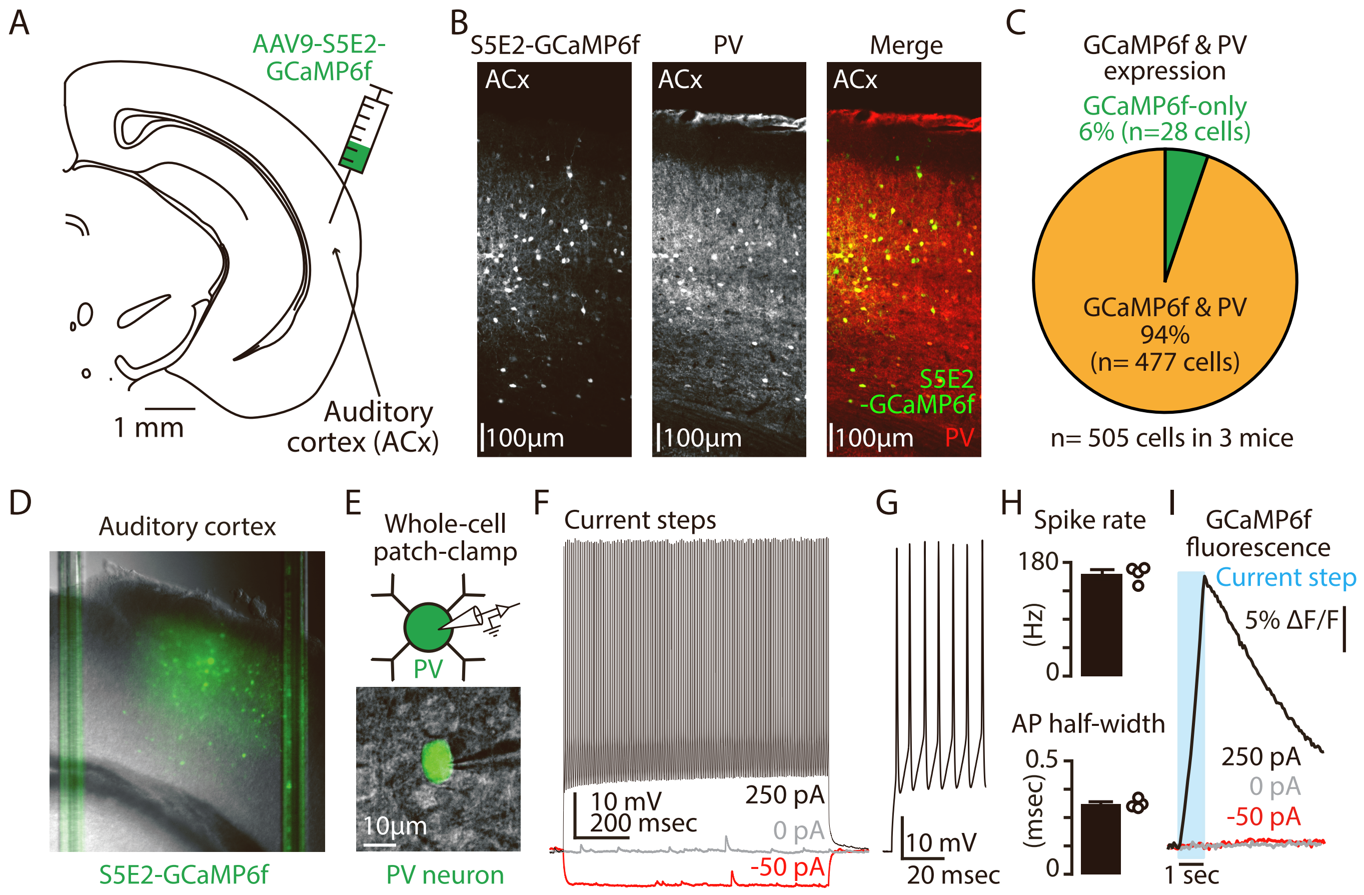

Fig. S4: AAV with the PV neuron-specific promoter S5E2 preferentially labels parvalbumin-expressing neurons in the auditory cortex

(A) Cartoon showing the stereotaxic injection scheme for expressing the genetically encoded fluorescent calcium sensor GCaMP6f in putative parvalbumin-expressing neurons using an AAV with the S5E2 promoter.

(B) Left: Example brain section in auditory cortex showing S5E2-promoter mediated expression of GCaMP6f in putative parvalbumin-expressing neurons. Center: The same acute section with neurons labeled by a parvalbumin antibody. Right: Merge of the GCaMP6f (green) and parvalbumin antibody (red) stains. Scale bar 100 μm.

(C) Pie chart showing that ~95 % of cells that were GCaMP6f-positive were also PV-positive (red and green) and ~5 % of cells were GCaMP6f -positive but not PV-positive (green).

(D) Acute brain slice image of a mouse auditory cortex with S5E2-mediated GCaMP6f expressing neurons (green).

(E) Top: schematic of a S5E2-mediated GCaMP6f expressing putative parvalbumin-expressing neuron (green) in whole-cell patch clamp configuration. Bottom: example image of a S5E2-mediated GCaMP6f expressing putative parvalbumin-expressing neuron (green) in whole-cell patch clamp configuration.

(F) Example current clamp recording of an S5E2-mediated GCaMP6f-expressing neuron at -50 pA (red), 0 pA (grey) and +250 pA (black) current steps showing membrane potential changes and neuronal spiking.

(G) Inset of the +250 pA current step current-clamp recording in (F) showing fast spiking rates.

(H) Top: Bar graph showing the average spike rate of putative parvalbumin interneurons expressing GCaMP6f via the S5E2 promoter recorded using whole-cell patch clamp. Bottom: Bar graph showing the average actional potential half-width of putative parvalbumin interneurons expressing GCaMP6f via the S5E2 promoter recorded using whole-cell patch clamp.

(I) Example GCaMP6f-mediated calcium fluorescence response to a 1 sec duration current step (blue) at -50 pA (red), 0 pA (grey), and +250 pA (black).

Asterisks indicate significant p values. Data are represented as mean ± SEM. See Table S1 for detailed statistics.

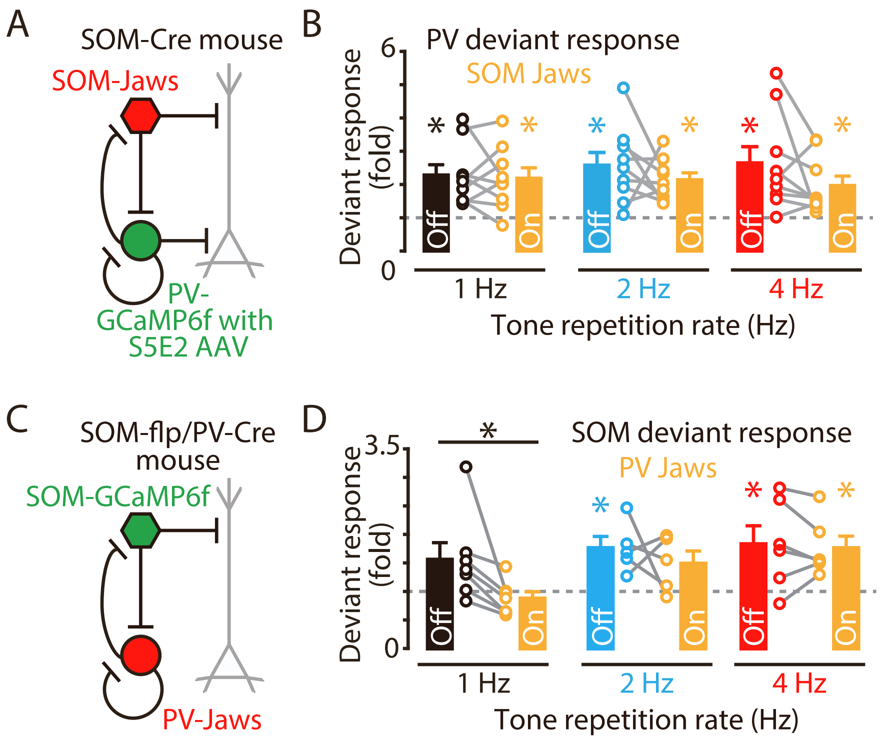

Fig. S5: Deviance detection by PV and SOM neurons during disinhibitory optogenetic experiments

(A) Cartoon circuit diagram showing Jaws-expressing SOM neurons (red) and GCaMP6f-expressing PV neurons (green).

(B) Responses of PV neurons to a deviant tone after a train of ten standard tones at different tone repetition rates. Average difference between the fluorescence response amplitude of the tenth (last) standard tone (grey dashed line) and the fluorescence response amplitude to the deviant tone in the stimulus train at in light-off controls and light-on inactivation of SOM neurons at 1 Hz (light-off: black, light-on: yellow), 2 Hz (light-off: blue, light-on: yellow), and 4 Hz (light-on: red, light-on: yellow) tone repetition rates (1 Hz; light-off: $p = 0.0032$, light-on: $p = 0.0055$; one-sample t-tests, light-off v. light-on: $p = 0.7465$, $n = 9$ recordings from 6 mice; paired t-test. 2 Hz; light-off: $p = 0.0032$, light-on: $p = 6.046e-4$; one-sample t-tests, light-off v. light-on: $p = 0.3586$, $n = 9$ recordings from 6 mice; paired t-test. 4 Hz; light-off: $p = 0.0097$, light-on: $p = 0.0092$; one-sample t-tests, light-off v. light-on: $p = 0.1757$, $n = 9$ recordings from 6 mice; paired t-test.)

(C) Cartoon circuit diagram showing Jaws-expressing PV neurons (red) and GCaMP6f-expressing SOM neurons (green).

(D) Responses of SOM neurons to a deviant tone after a train of ten standard tones at different tone repetition rates. Average difference between the fluorescence response amplitude of the tenth (last) standard tone (grey dashed line) and the fluorescence response amplitude to the deviant tone in the stimulus train at in light-off controls and light-on inactivation of SOM neurons at 1 Hz (light-off: black, light-on: yellow), 2 Hz (light-off: blue, light-on: yellow), and 4 Hz (light-off: red, light-on: yellow) tone repetition rates (1 Hz; light-off: $p = 0.0998$, light-on: $p = 0.3471$; one-sample t-tests, light-off v. light-on: $p = 0.0377$, $n = 7$ recordings from 4 mice; paired t-test. 2 Hz; light-off: $p = 0.0177$, light-on: $p = 0832$; one-sample t-tests, light-off v. light-on: $p = 0.5116$, $n = 5$ recordings from 3 mice; paired t-test. 4 Hz; light-off: $p = 0.0466$, light-on: $p = 0.0136$; one-sample t-tests, light-off v. light-on: $p = 0.6829$, $n = 6$ recordings from 3 mice; paired t-test.)

Asterisks indicate significant p values. Data are represented as mean ± SEM. See Table S1 for detailed statistics.
